# Oxidative Stress and Energy Metabolism in the Brain: Midlife as a Turning Point

**DOI:** 10.3390/antiox10111715

**Published:** 2021-10-28

**Authors:** Volodymyr I. Lushchak, Michael Duszenko, Dmytro V. Gospodaryov, Olga Garaschuk

**Affiliations:** 1 Department of Biochemistry and Biotechnology, Vasyl Stefanyk Precarpathian National University, 57 Shevchenko Str., 76018 Ivano-Frankivsk, Ukraine; volodymyr.lushchak@pnu.edu.ua (V.I.L.); dmytro.gospodaryov@pnu.edu.ua (D.V.G.); 2 Department of Medical Biochemistry, I. Horbachevsky Ternopil National Medical University, 46002 Ternopil, Ukraine; 3 Research and Development University, 13a Shota Rustaveli Str., 76018 Ivano-Frankivsk, Ukraine; 4 Department of Neurophysiology, Institute of Physiology, University of Tübingen, 72074 Tübingen, Germany; midu@live.de

**Keywords:** reactive oxygen species (ROS), reactive nitrogen species (ROS), midlife, aging, inflammation, redox metabolism, mitochondria, microglia

## Abstract

Neural tissue is one of the main oxygen consumers in the mammalian body, and a plentitude of metabolic as well as signaling processes within the brain is accompanied by the generation of reactive oxygen (ROS) and nitrogen (RNS) species. Besides the important signaling roles, both ROS and RNS can damage/modify the self-derived cellular components thus promoting neuroinflammation and oxidative stress. While previously, the latter processes were thought to progress linearly with age, newer data point to midlife as a critical turning point. Here, we describe (i) the main pathways leading to ROS/RNS generation within the brain, (ii) the main defense systems for their neutralization and (iii) summarize the recent literature about considerable changes in the energy/ROS homeostasis as well as activation state of the brain’s immune system at midlife. Finally, we discuss the role of calorie restriction as a readily available and cost-efficient antiaging and antioxidant lifestyle intervention.

## 1. Introduction

Living organisms are open thermodynamic systems, critically relying on their energy metabolism for the maintenance of structural integrity and function. Energy homeostasis includes catabolic processes that start with the degradation of polysaccharides, lipids and proteins and lead to the production of energy-reach compounds. Adenosine triphosphate (ATP), the universal macroergic compound used by living organisms, is produced via multistep anaerobic or aerobic oxidation processes.

**Figure 1:**
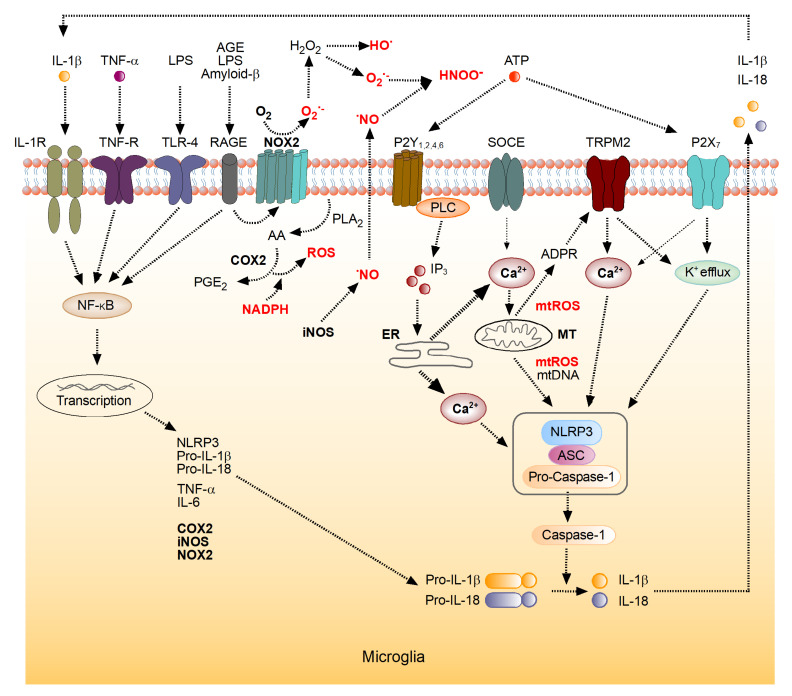
Molecular mechanisms underlying microglial reactive oxygen species (ROS) metabolism. Mechanisms involved in the interplay between the inflammation-mediated ROS production, increases in [Ca^2+^]_i_ and cytokine production by microglia (see text for detailed description). IL-1β, interleukin 1β; TNF-α, tumor necrosis factor α; LPS, lipopolysaccharide; AGE, advanced glycation endproducts; IL-1R, IL-1β receptor; TNF-R, TNF-α receptor; TLR-4, Toll-Like Receptor 4; RAGE, a receptor for AGE; NADPH, a reduced form of nicotinamide adenine dinucleotide phosphate; NOX2, NADPH oxidase 2; P2Y_1,2,4,6_, metabotropic ATP receptors, SOCE, store-operated Ca^2+^ entry channel; TRPM2, Transient receptor potential cation channel, subfamily M, member 2; P2X_7_, ionotropic ATP receptor, PLA_2_, phospholipase A2; AA, arachidonic acid; COX2, cyclooxygenase 2; PGE_2_, prostaglandin E2; iNOS, inducible NO-Synthase; PLC, Phospholipase C; IP_3_, inositol 1,4,5-trisphosphate; ER, endoplasmic reticulum; MT, mitochondrion; mtROS, reactive oxygen species of mitochondrial origin; mtDNA, mitochondrial DNA; ADPR, ADP-ribose. The boxed structure consisting of NLRP3, adaptor protein ASC and Pro-Caspase-1 represents the NLRP3 inflammasome (modified from [14,16]).

**Figure 2:**
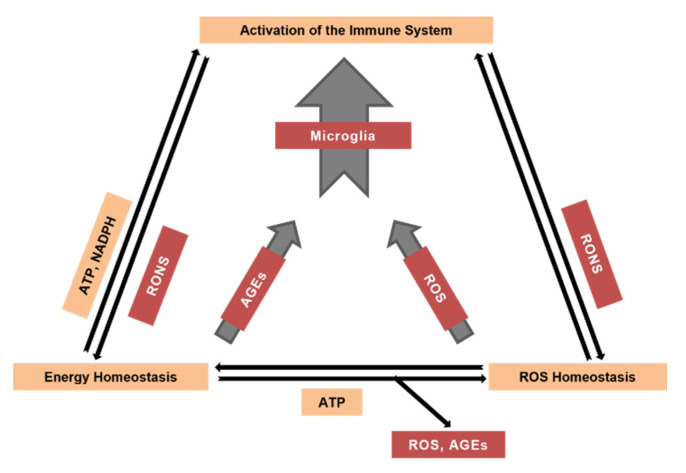
The interplay between the energy/ROS homeostasis and the activation of the brain’s immune system during aging. RONS, reactive oxygen/nitrogen species.

Aerobic ATP production is accompanied by the generation of reactive oxygen species (ROS) as side products of operation of the mitochondrial electron transport chain [1,2]. Thus, besides producing energy-reach substrates, the energy homeostasis generates potentially damaging side products such as ROS in the respiratory chain and methylglyoxal in glycolysis. Methylglyoxal and other α-dicarbonyl compounds can react with arginine and lysine residues of proteins, yielding glycated derivatives called advanced glycation end products (AGEs), while ROS can interact with virtually all components of living organisms, causing their modification [3,4,5]. ROS are also produced by the metabolic systems not connected with energy production. In particular, monoamine oxidases, cytochromes P450, peroxisomal oxidases and plasma membrane-bound Nicotinamide adenine dinucleotide phosphate (NADPH) oxidases (NOX) can produce ROS [6]. Since ROS discovery in living organisms in the early 1950th, they have been considered as damaging species [7]. Therefore, most ROS studies focused on the investigation of their negative effects and protection against them [8]. However, besides the damaging effects, at physiological concentrations mitochondrial and cytosolic ROS play important signaling roles in multiple cellular processes, including inflammation, cellular growth and differentiation [9,10,11,12]. Whether ROS effects are beneficial or detrimental depends on the balance between ROS generation and elimination as well as on the targets attacked [11]. Usually, the antioxidant system of a young organism copes with oxidative modifications of biomolecules but gradually loses this ability during aging. Therefore, aging is accompanied by an increase in oxidative stress, a decreased efficiency of ATP production and the concomitant activation of the immune system [3,4,13], because both ROS and AGEs activate key proinflammatory molecules nuclear factor kappa-light-chain-enhancer of activated B cells (NF-κB) and NLR Family Pyrin Domain Containing 3 (NLRP3) (Figure 1), causing enhanced production of proinflammatory cytokines [14,15,16]. Inflammation, triggered in this way, leads to the production of different reactive species, particularly ROS (Figure 1), thus feeding back to energy and ROS homeostasis (Figure 2) [17,18]. While in a young organism byproducts generated by the energy metabolism are efficiently neutralized by the respective defense systems, these processes become imbalanced with advancing age [4,19].

The brain is especially prone to ROS-mediated toxicity for several reasons: (i) the intense oxidative metabolism, (ii) the high levels of polyunsaturated fatty acids, serving as primary substrates for ROS-promoted oxidation and (iii) a rather high number of resident immune cells [20]. Recently, we discovered that crucial changes in the functional properties of microglia, the brain’s resident immune cells, as well as the energy and redox metabolisms, occur already at middle age [4,21,22,23,24,25]. Here, we discuss the interplay between the energy and redox metabolism of the brain and its immune system along the lifespan and focus on middle age as a critical point for the reorganization of energy and redox homeostasis as well as the operation of the brain’s immune system.

## 2. Energy Metabolism of the Brain

The brain contributes only 2% to body weight but consumes about 15% of cardiac output, about 25% of total glucose and some 20% of total oxygen utilized by the organism [26]. It carries out an intense oxidative metabolism, which is on average ~10 times higher than in the rest of the body. In the brain, energy is needed not only for the homeostasis of cell metabolism but especially for neuronal activity/plasticity including maintenance and restoration of the membrane potential following depolarization, the synthesis/re-utilization of neurotransmitters, and for intracellular trafficking of vesicles from Golgi apparatus to the synapse and vesicle recycling. The energy need of the brain depends on the strength of neuronal activity. The different demand for oxygen and metabolites to support active neurons is paralleled by a regulated blood supply depending on vascular constriction or dilation. In addition, energy metabolism is significantly different in specified brain cells. Neurons consume up to 80% of the energy produced in the brain [27]. Therefore, a well-balanced and meticulously controlled regulation of the energy metabolism in the brain is a prerequisite for efficient neuronal functioning.

Since neuronal activity depends on a sufficient supply of energy substrates (mainly glucose) and O_2_, local cerebral blood flow increases upon an increase in neuronal activity (functional hyperemia) due to neurovascular coupling [28]. The interface for metabolite exchange is the blood-brain barrier (BBB). This barrier is formed by endothelial cells interconnected by tight junctions and lining the blood vessels together with a basal membrane surrounding the vessels. In addition, the surface capillaries are covered by astrocyte endfeet (about 80%) and pericytes (about 20%), with the latter controlling the blood flux by dynamic cell size variation. To ensure a sufficient supply of energy substrates, the total length of vessels in the adult human brain equals some 600 km [29]. Interestingly, fatty acids—a major energy substrate for other tissues—seem not generally being used by brain cells, although the brain possesses a relatively high lipid content (about 60%) and contains more long-chain polyunsaturated fatty acids than any other tissue [30]. After the adaptation of brain cells (usually lasting 2–3 weeks), ketone bodies, formed by hepatocytes during an extended period of starvation, may be used to replace about 50% of the glucose need. In contrast, a hypoglycemic shock, i.e., a glucose level below 3 mmol/L, may lead within 30 min to coma and death at a level below 1.5 mmol/L; block of oxygen supply leads to unconsciousness within seconds and irreversible damages of neurons after minutes.

Glucose from the blood enters the interstitium by transcellular transport across endothelial cells using glucose transporter 1 (GLUT1) and is taken up primarily via the astrocytic GLUT1 transporter. Energy metabolism in these cells is primarily but not exclusively driven by aerobic glycolysis, meaning conversion of glucose to pyruvate. However, in contrast to erythrocytes where mitochondria are absent and lactate is formed to re-oxidize NADH, thus avoiding a shortage of redox equivalents, lactate is formed because pyruvate dehydrogenase (PDH) is mostly phosphorylated in these cells and thus is in its inactive state [31]. Thus, astrocytes have a high glycolytic rate, leading inevitably to the formation of methylglyoxal due to non-enzymatic dephosphorylation of glyceraldehyde-3-phosphate and dihydroxyacetone phosphate. Interestingly, astrocytes express increased levels of glyoxalases to detoxify cytotoxic methylglyoxal [32]. In case of high energy demand, PDH may be activated to convert pyruvate to acetyl-coenzyme A (acetyl-CoA), thus fueling the Krebs or tricarboxylic acid (TCA) cycle for ATP generation by oxidative phosphorylation. In this context, transport mechanisms for the uptake of free fatty acids from the blood and their oxidation, especially in astrocytes have been described (reviewed in [33]). Thus, astrocytic ATP production is not exclusively dependent on glycolysis. In fact, glycolytic metabolites like glucose-6-phosphate are also needed for glycogen biosynthesis as a glucose pool for urgent energy need or to fuel the pentose phosphate pathway (PPP) for NADPH and ribose production, while glyceraldehyde-3-phosphate is used for serine formation as a precursor for glycine and cysteine production, both needed for glutathione biosynthesis as a first-line defense against ROS. Lactate, the end product of glycolysis in astrocytes, is secreted via monocarboxylate transporter (MCT) 4 and taken up by neurons via MCT2 transporter [34,35]. It is then converted by lactate dehydrogenase 1 (LDH1) to pyruvate that after conversion by PDH enters the TCA cycle as acetyl-CoA. This feeding of neurons by astrocytes (lactate shuttle) is nicely reflected by the distribution of LDH isoenzymes: LDH5 (conversion of pyruvate to lactate) in astrocytes and LDH1 (conversion of lactate to pyruvate) in neurons [36]. Astrocytes are not the exclusive source of lactate, however, as lactate can also be taken up from the blood via MTCs and may account for up to 25% of the neuronal energy substrate during high neuronal activity [37]. Thus, at least in active neurons energy is generated primarily via mitochondrial oxidative phosphorylation driven by redox equivalents from the TCA cycle and molecular oxygen. The critical role of mitochondria for brain’s energy provision is underscored by the fact that mutations in genes encoding mitochondrial proteins often lead to encephalopathies and neurodegeneration [38]. Age-dependent neurodegeneration is also associated with an impairment of mitochondrial function [39]. For instance, an administration of rotenone, an inhibitor of complex I (NADH:ubiquinone oxidoreductase) of the mitochondrial respiratory chain, leads to the development of parkinsonian symptoms in rats [40].

Accordingly, glycolysis is decreased in neurons due to the constant degradation of phosphofructokinase 2 (PFK2) by proteasomes [41]. PFK2 is the most powerful regulator of glycolysis known to date. This bifunctional enzyme possesses a kinase activity to phosphorylate fructose-6-phosphate to fructose-2,6-bisphosphate and a phosphatase activity to decrease the concentration of fructose-2,6-bisphosphate. Since fructose-2,6-bisphosphate activates PFK1 and thus glycolysis, a markedly decreased PFK2 activity leads to the increased steady-state level of glucose-6-phosphate that then fuels the PPP leading primarily to the formation of NADPH that is needed to re-oxidize reduced glutathione via glutathione reductase. Glutathione is needed to cope with high ROS production in active neurons.

Besides lactate, molecular oxygen is needed for ATP production via oxidative phosphorylation. Bound to hemoglobin, O_2_ appears in brain vessels and depending on the oxygen gradient diffuses across membranes into brain cells. In active neurons, the oxygen uptake is especially high. To support this, blood supply must be increased. Partially, the local blood vessel’s diameter increases due to dilation of pericytes, mediated by the activation of K^+^ channels, hyperpolarization of the cell membrane and the closure of voltage-gated Ca^2+^ channels [42]. In addition, neurons and endothelial cells produce vasoactive agents, including ^•^NO, generated by the endothelial nitric oxide synthase (eNOS), prostaglandin E_2_ (PGE2) and epoxyeicosatrienoic acids (EETs) [43,44]. Neuronal firing, the subsequent neurotransmitter release, and the activation of postsynaptic N-methyl-D-aspartate (NMDA) receptors by glutamate also activates the neural NO synthase (nNOS) and thus the formation of the free radical nitric oxide (^•^NO) from arginine and O_2_. Dilation of pericytes is primarily induced by PGE2 but is supported by ^•^NO-mediated inhibition of the synthesis of the vasoconstrictory 20-hydroxyeicosatetraenoic acid (20-HETE) [45]. On the contrary, an increased blood flow seems to decrease the ^•^NO concentration, as ^•^NO is scavenged by erythrocytes [46].

There is no question that oxygen is indispensable for neuronal activity, it is technically difficult though to define a minimum oxygen concentration beyond which cellular damages occur. For the adult rat brain, a concentration below 8 mm Hg seems to be critical [47], while a low oxygen partial pressure seems necessary for the embryonic brain to develop normally [48], and hypoxia during stroke was shown to induce neuronal stem cell proliferation [49]. Here, hypoxia-inducible factors (HIFs) seem to link oxygen tension to gene expression [50]. Especially the HIF-1α subunit of the HIF-1α/β heterodimer seems to be important. It is synthesized constitutively but under oxidative conditions is subjected (likely in a ROS-dependent manner) to constant ubiquitinoylation and proteasomal degradation. Under hypoxia-induced reducing conditions, however, HIF-1α is stable and works as a transcription factor (reviewed by [51]).

## 3. Mechanisms of ROS Generation

The bulk ROS amount in the organisms is generated by poorly controlled non-enzymatic processes such as autoxidation of small molecules (e.g., epinephrine, quinones), by better controlled enzymatic processes (e.g., operation of different oxidases) and by the escape of active electrons from electron transport chains [11]. Under normoxic conditions, more than 90% ROS in eukaryotic cells are generated by mitochondria [52], where above 95% of consumed oxygen is reduced via four-electron reduction of molecular oxygen by cytochrome oxidase [53]. During the operation of mitochondria, however, some of the transported electrons escape ETC and join molecular oxygen giving rise to ROS. Besides the respiratory chain, ROS can be generated by multi-enzyme flavin-containing complexes such as alpha-ketoglutarate dehydrogenase and pyruvate dehydrogenase. Other enzymatic generators of ROS are monoamine oxidases located in the mitochondrial outer membrane. The latter way of ROS formation is especially important for neurons that produce and secrete biogenic amines [6,38]. Brain cyclooxygenases, lipoxygenases and NADPH oxidases also generate ROS. NADPH oxidases (NOXs) are often associated with immune cells like neutrophils and macrophages. However, they are also expressed in brain vascular endothelium and microglia and may contribute to age-related changes [12,14,54]. These enzymes specifically produce either superoxide anion radical (O_2_^•−^) or hydrogen peroxide (H_2_O_2_) or both, transferring electrons from NADPH to flavin adenine dinucleotide (FAD) first, and then—to heme of cytochrome *b*_558_, and—from cytochrome *b*_558_ to oxygen [55]. Cyclooxygenases are heme-containing enzymes that convert arachidonic acid to prostaglandin H2. These enzymes can produce O_2_^•−^ since the catalysis occurs via the formation of transient carbon-centered radical species that, in turn, can react with oxygen. Importantly, the formation of O_2_^•−^ by cyclooxygenases requires reducing co-substrates such as NADH or NADPH [56,57]. Superoxide is also produced in the brain endothelial cells by xanthine oxidase, a peroxisomal flavin-containing enzyme [58].

In most cases where ROS are formed by enzymatic systems, sequential multistep one-electron O_2_ reduction takes place. Interaction of one electron with molecular oxygen results in the formation of O_2_^•−^. The latter can interact with one more electron and two protons forming H_2_O_2_. At the next reduction step, H_2_O_2_ can accept one more electron, resulting in the production of hydroxyl radical (HO^•^) and hydroxyl anion (OH^−^). Finally, HO^•^ may accept another electron and one proton, while OH^−^ may associate with a proton; in both cases water molecules are formed. All three partially reduced oxygen intermediates, namely free radicals O_2_^•−^ and HO^•^ as well as H_2_O_2_ are called reactive oxygen species because they are more active than molecular triplet oxygen [2,59]. ROS-induced reactions occur spontaneously and are poorly controlled by living organisms.

All cells with aerobic metabolism possess a set of low and high molecular mass antioxidants [11]. Low molecular mass antioxidants such as vitamins C and E, carotenoids, anthocyans and glutathione interact directly with any ROS type at low specificity. However, biological protection against HO^•^ is difficult, probably due to its high chemical activity, short lifespan and small diffusion distance, and thus organisms try to prevent HO^•^ production. In terms of high molecular mass antioxidants, levels of O_2_^•−^ and H_2_O_2_ are also controlled by enzymatic systems. Antioxidant enzymes form the so-called primary line, which directly deals with ROS, and a secondary line, which assists to the primary one and converts ROS-related components to less dangerous products. In addition, several enzymes regenerate low molecular mass antioxidants and repair certain types of oxidative damage [6,11]. Superoxide dismutases accelerate the conversion of O_2_^•−^ in H_2_O_2_ with concomitant formation of molecular oxygen, i.e., they dismutate one type of substrate molecules in two different products. Hydrogen peroxide can be eliminated by two types of enzymes: catalase, which dismutates H_2_O_2_ to water and molecular oxygen, and peroxidases, which use diverse co-substrates to reduce H_2_O_2_ to water and the respective oxidized co-substrate. Manganese-containing superoxide dismutase (Mn-SOD), specific glutaredoxins (Grx5), thioredoxins and peroxiredoxins (Prdx3 and Prdx5) scavenge ROS released to both, mitochondrial matrix and intermembrane space [60]. In turn, O_2_^•−^ released by non-mitochondrial sources, such as NOX, cytochromes P450 and peroxisomal oxidases (for instance, xanthine oxidase) is scavenged by cytosolic copper-zinc-containing superoxide dismutase (Cu,Zn-SOD). Hydrogen peroxide, which is small and uncharged molecule, can easily cross lipid membranes. Whatever the place of H_2_O_2_ formation, it has a great chance to occur in the cytosol where it can be converted to water by peroxidases. Peroxidases use different cofactors and glutathione peroxidases are likely the best-studied enzymes of this group [61]. They use the reductive power of glutathione that is oxidized to a dimeric form and may be further reduced by glutathione reductase at the expense of NADPH. The formed NADP^+^ is then reduced by glucose-6-phosphate dehydrogenase, the key enzyme of the pentose phosphate pathway, that oxidizes glucose-6-phosphate to 6-phosphoglucolactone. This reaction finally connects ROS homeostasis with the catabolism of carbohydrates and overall energy-providing processes (Figure 2). An imbalance between ROS generation and detoxification will eventually enhance the oxidation of important biomolecules and induce a loss of their functions. The systems responsible for the antioxidant defense, i.e., the elimination of oxidized molecules either by repair or biosynthesis wear out with cell’s age. This is especially relevant for neural tissue, which is predominantly composed of cells that rarely divide or regenerate.

## 4. The Role of the Brain’s Immune System in the Generation of ROS

Microglia, the main immune cells of the brain, vividly utilize ROS-mediated signaling under (patho)physiological conditions, and, consequently, possess several mechanisms for the generation of both intra- and extracellular ROS [12,14,15]. As illustrated in Figure 1, microglia express NADPH oxidases, capable of generating O_2_^•−^ and H_2_O_2_. Recent single-cell RNA sequencing analyses identified NADPH oxidase NOX2 as an isotype with the highest expression level in both human and mouse microglia [12,14]. In both species, the robust expression of NOX2 was seen not only during adulthood but also during development. NOX4 was also expressed in microglia, albeit at a much lower level than NOX2 [12]. Besides NOX, NO synthases and cyclooxygenases are relevant sources of microglial ROS [14]. For example, activation of microglia is associated with an NF-κB-dependent upregulation of iNOS and COX2 expression and a concomitant overproduction of intracellular ROS (Figure 1). The intracellularly generated ^•^NO diffuses out of the cell and, besides acting as a secondary messenger, reacts with superoxide anion, generated by NOX2, forming peroxynitrite (ONOO^−^). The latter is a highly reactive nitrogen species often causing tissue, cell and mitochondrial damage [12,62].

Like many other non-excitable cells, microglia utilize changes in the intracellular free Ca^2+^ concentration ([Ca^2+^]_i_) for executing their sensor and effector functions [63,64,65]. Such Ca^2+^ signaling, mediated, for example, by activation of a plentitude of metabotropic receptors or store-operated Ca^2+^ channels (Figure 1) causes a release of ROS from mitochondria [16,66]. In turn, ROS produced in cytosol or mitochondria increase the production of ADP-ribose through degradation of poly-ADP-ribose in the nucleus or degradation of NAD^+^ released from damaged mitochondria in the cytoplasm [66,67]. Together, ADP-ribose and Ca^2+^ activate Ca^2+^-permeant Transient Receptor Potential (TRPM2) channels (Figure 1), known for their sensitivity to endogenous ROS [67], thus further increasing [Ca^2+^]_i_. In this way, TRPM2 channels link ROS production to inflammasome activation in immune cells, where the expression of these channels is abundant.

## 5. Midlife Turning Point in Glucose Catabolism: Switch from Glycolysis to Pentose Phosphate Pathway

As already mentioned above, aerobic ATP production is accompanied by ROS formation and redox processes. ROS-induced peroxidation of polyunsaturated fatty acids produces lipid hydroperoxides (LOOH), which are frequently used as biomarkers of oxidative stress [2]. Despite an intensive oxidative metabolism, no highly efficient brain-specific ROS defense systems were described so far. Surprisingly, however, the levels of ROS-mediated lipid/protein oxidation products in the brain are comparable to those found in other organs [25,68].

**Figure 3:**
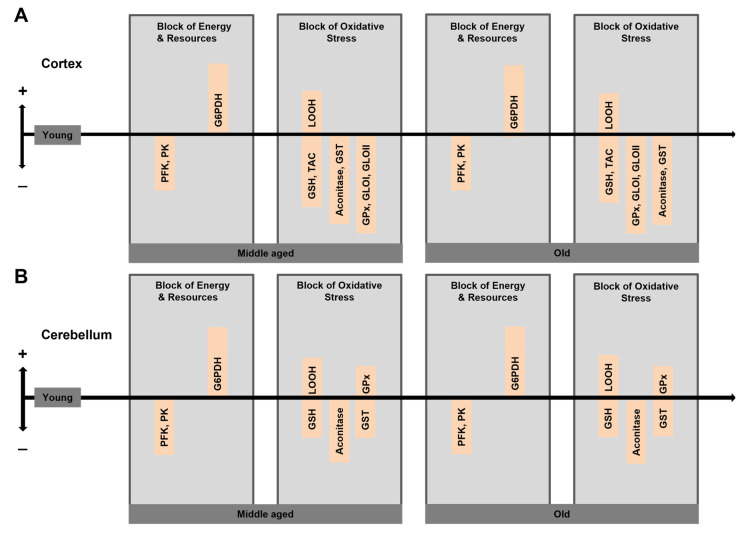
Aging-related changes in the energy homeostasis and the redox state of the brain. Summary of data from the mouse cerebral cortex (**A**) and cerebellum (**B**). Deduced from [24,25].

Generally, brain aging is associated with enhanced steady-state levels of ROS-oxidized compounds and their complexes [3]. There are, however, also some conflicting literature data finding no significant difference in levels of ROS-modified components as compared to other markers of oxidative stress between old and younger animals. To clarify this issue, we designed an experiment with three age groups: young adult (6-month-old), middle-aged (12-month-old) and old (18-month-old) mice. The results obtained [24] showed for the cerebral cortex that levels of oxidative stress markers, such as (LOOH), were higher in middle-aged compared to young mice, whereas the levels of glutathione (GSH) and total antioxidant capacity (TAC) were lower (Figure 3A,). Unexpectedly, these parameters were similar between old and middle-aged mice. The data obtained in the cerebellum largely confirmed the cortical data (Figure 3B,). Interestingly, virtually no differences in age-related changes were found between males and females. Since age-related profiles may differ for different markers of oxidative stress [3,4], when comparing the aged group with the younger cohort [24,25] the results obtained depend on at least two conditions: (1) the relative age of the younger group and (2) the nature of parameters under study.

Our data suggest that the mechanisms, counteracting the age-related intensification of oxidative stress, include the upregulation of PPP and concomitant downregulation of glycolysis [24]. As a consequence, energy metabolism seems to change throughout the lifespan with glucose catabolite fluxes being redistributed with advanced age between glycolysis and PPP, in favor of the latter [3]. According to our rodent data, principal changes in the energetic homeostasis take place already at the transition between young and middle age, whereas the difference between middle and old age is minimal. This redistribution of the glucose intermediate fluxes between glycolysis and PPP may represent an efficient mechanism to strengthen the antioxidant defense and to prevent further intensification of oxidative stress. In our recent work, we have disclosed some molecular mechanisms underlying such changes [24,25]. In both, cortex and cerebellum, the activities of key glycolytic enzymes PFK and PK were lower in middle-aged and old mice relative to young ones (Figure 3). At the same time, the activity glucose-6-phosphate dehydrogenase, a key enzyme of PPP, was substantially upregulated compared to the activity in young animals (Figure 3). Such changes form the molecular basis for the potential strengthening of the antioxidant system during aging, because NADPH produced by PPP may be used by the antioxidant system to combat ROS. Note, however, that the cells, which do express NOX2 (e.g., microglia) might use NADPH to generate ROS.

Which mechanisms are responsible for the age-related decrease in activities of key glycolytic enzymes and redirection of carbohydrate metabolism from glycolysis to PPP in mouse cortex and cerebellum? Several systems are known to regulate the organism’s response and adaptation to oxidative stress, with the key role of the Nrf2/Keap1 system [2,3,4,11,69,70]. Reversible oxidation or electrophilic attack on certain cysteine residues of sensor proteins is the primary redox signaling process. This signal targets molecular regulatory machines. In a reduced state Keap1, a negative regulator of transcriptional factor Nrf2 (nuclear factor erythroid-derived 2-like factor 2), binds Nrf2 and promotes its subsequent ubiquitination [2,70]. Under oxidative stress, redox-sensitive cysteine residues of Keap1 are oxidized by ROS leading to a conformational change of the protein. This change precludes Keap1 binding to Nrf2 protein. Nrf2 migrates into the nucleus where it upregulates the expression of about 200 genes [71]. Protein products of some Nrf2 target genes are clearly protective against ROS (e.g., SOD, thioredoxins and thioredoxin reductases). Other Nrf2 target genes encode proteins responsible for biosynthesis and reduction of low molecular mass antioxidants (e.g., glutathione reductase), xenobiotic detoxification (for instance, by glutathione-*S*-transferases and UDP-glucoronosyl transferases) [70].

In our studies, this explanation worked fine when comparing oxidative stress parameters between young and middle-aged mice [24,25], but did not explain why similar data were obtained in middle-aged and old mice. Despite the general belief that the intensity of oxidative stress is enhanced during aging, the data on the oxidative state of the cysteome (or redoxome) during lifespan are contradictive [72,73,74]. The Redox theory of aging relates aging to a decline in a plastic interaction between genome and environment, thus causing many hallmarks of aging, particularly the failure to maintain oxidative defense [61]. This theory was further extended to explain the loss of adaptive homeostasis in concert with hormetic responses to various stresses [75]. The authors summarized data from many laboratories and concluded that in the nematode *Caenorhabditis elegans*, the fruit fly *Drosophila melanogaster* and in mice an age-dependent loss of adaptive homeostasis takes place. The treatment by oxidants was a common denominator in the analyzed studies. This may suggest the involvement of Nrf2-sensor protein via reversible oxidation of some of its cysteine residues. These conclusions were supported by a nice work in *C. elegans* showing that so-called “Redox-stress response capacity” decays in an age-dependent way [76].

## 6. Midlife Increase in Mitochondrial Function Followed by Its Subsequent Decline

It is assumed that aging is accompanied by a continuous and progressive decline in mitochondrial metabolic activity. However, recent data from yeast, worms, flies, mice and monkeys, support a biphasic alteration in metabolic activity and mitochondrial function with age [77]. In particular, it was shown that mitochondrial metabolism, i.e., respiration, tricarboxylic acid cycle and acetyl-CoA synthesis, increases between young and middle-aged animals, with a subsequent decline towards old age. According to the biphasic model, the middle-age increase in mitochondrial activity may promote various types of cellular damage (e.g., oxidative stress), and may increase ROS levels [77]. Our recent study also supports this model as we have observed an increase in aconitase activity, mitochondrial complexes I and IV (cytochrome *c* oxidase) in middle-aged *ad libitum* fed male mice [24]. On the other hand, several studies conducted in different experimental models demonstrate a gradual decline of mitochondrial function with age [78,79,80,81,82,83]. Li et al. transferred freshly isolated mitochondria from brain synaptosomes to cells lacking mitochondrial DNA to generate cybrids, exclusively containing brain mitochondrial DNA from young, middle-aged and old C57BL/6 mice and found a gradual loss of mitochondrial complex I-dependent respiration with age [82]. Cybrids from the brains of old mice had a lower rate of uncoupled respiration than those from the brains of young and middle-aged individuals [82]. Consistent with this, our results show a gradual decline in ATP synthase activity with age in brain mitochondria of *ad libitum* fed mice [24]. A similar trend was observed by Gauba and colleagues [83]. In contrast, a gradual increase in ATP synthase activity with age was observed for intermittently fasted mice [24]. Another study showed a significant age-dependent drop in the activities of pyruvate dehydrogenase and cytochrome *c* oxidase [81]. Noteworthy, the activity of cytochrome *c* oxidase in non-transgenic C57BL6/129S females significantly decreased after 9th month of age. Simultaneously, there was an age-dependent increase in the expression of long-chain hydroxyacyl-CoA-dehydrogenase and 3-oxoacid-CoA transferase 1 [81]. Both enzymes allow cells to utilize ketone bodies as an alternative to glucose and/or pyruvate energy sources by converting them into acetyl-CoA. The shift to ketone body metabolism in the aging brain is supposedly connected with activation of sphingomyelinase by hydrogen peroxide causing age-dependent demyelination of axons in female mice. In turn, astrocytes use myelin as a source of fatty acids that are subsequently metabolized to ketone bodies [84]. Further, ketone bodies are transported from astrocytes to neurons where they are used as an energy source in parallel with glucose. As already mentioned above, fasting can stimulate ketone body production via catabolism of lipids stored in other organs such as adipocytes and hepatocytes. Some dietary interventions, i.e., ketogenic diet, can increase the level of ketone bodies in the blood. It was shown that utilization of ketone bodies as an energy source by neurons may improve brain health (reviewed in [85]). In particular, in neurons ketone bodies were shown to promote mitochondrial biogenesis mediated by the brain-derived neurotrophic factor (BDNF). In turn, the activity of BDNF is specifically regulated by ketone body *β*-hydroxybutyrate that was shown to inhibit deacetylases, which inactivate BDNF [86].

**Figure 4:**
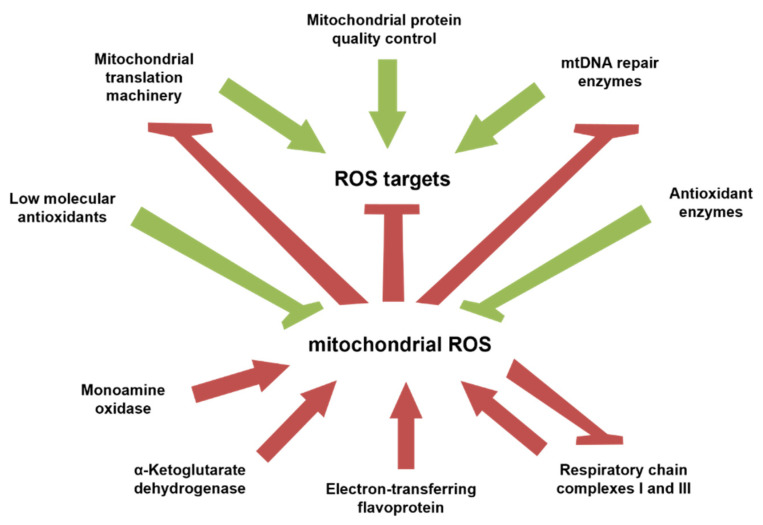
Relationship between pro- and antiaging processes and mitochondrially produced ROS that may account for the rapid decline of mitochondrial function in the second half of life. Red activation and inhibition arrows denote pro-aging players, whereas green activation and inhibition arrows denote antiaging players.

Besides, all age-dependent changes occur on the background of ROS-induced damage of cellular components on the one hand and repair/elimination as well as *de novo* biosynthesis of damaged components on the other hand [87]. In turn, dysfunctional respiratory chain complexes often augment ROS generation [6]. Mutations in genes encoding either subunits of mitochondrial respiratory chain complexes or enzymes responsible for antioxidant defense might pre-dispose an organism to pathology and hasten the onset of age-related symptoms [39]. Thus, improperly assembled respiratory chain complexes, including those lacking prosthetic groups, produce ROS at higher rates than the intact complexes [88,89,90,91]. Moreover, respiratory chain complexes are combined in supercomplexes. The composition of these supercomplexes dramatically influences rates of ROS generation. In particular, it was demonstrated that the respiratory chain complex I is hardly included in supercomplexes upon knockdown of its subunit NDUFS1 [92]. This leads to a prevalence of free complex I and an increased generation of ROS. Thus, the effectiveness of repair processes and quality control for mitochondrial translation machinery; multi-protein complex assembly and biosynthesis of essential prosthetic groups, such as iron-sulfur clusters and heme, would determine the intensity of ROS generation. The senescence, including that of the brain, is characterized by a gradual and relatively slow loss of mitochondrial functionality. ROS produced by the respiratory chain complexes and multiple flavin-containing enzymes mentioned above oxidize mitochondrial proteins, lipids and nitrogen bases of mitochondrial DNA. This process is slowed down by the antioxidant system, which intercepts and detoxifies ROS, and by a constantly operating mitochondrial translation machinery and DNA repair systems. However, the antioxidant and DNA repair enzymes as well as protein translation machinery are also ROS targets (Figure 4).

The increase in mitochondrial ROS production, caused by an age-dependent decline in the effectiveness of biosynthesis and repair processes, can be accelerated by elevated Ca^2+^ levels within the cells [16] and by advanced oxidation of mitochondrial proteins and lipids. It is believed that the most sensitive ROS targets of mitochondria are iron-sulfur clusters of aconitase [93], thiols of voltage-dependent anion channel (VDAC), also known as porin [94], pyruvate carboxylase, E1 component of pyruvate dehydrogenase, Mic60 or mitofillin [95]. Adenine nucleotide translocase, aspartate amino transferase, subunits NDUFS1 and NDFUS2 of mitochondrial respiratory chain complex I, subunits of cytochrome c oxidase and ATP synthase were found to be prone to modification by 4-hydroxynonenal, one of the main lipid peroxidation products [96,97]. Other proteins that are critically important for mitochondrial function, such as DNA polymerase γ, were also found to be sensitive to oxidation [98]. Mitochondrial proteins are encoded by both nuclear and mitochondrial genomes. Therefore, their *de novo* biosynthesis depends on the integrity of these genomes as well as translation machinery. Note that the rate of age-dependent tissue deterioration strongly depends on the DNA repair systems. Diseases associated with mutations in genes encoding DNA repair proteins, e.g., structure-specific endonuclease Ercc1, lead to premature aging [99]. The machinery responsible for DNA repair works reliably until late middle or old age [99]. Mitochondria contain their own DNA repair machinery, which is encoded by the nuclear genome [100]. Unlike nuclear DNA, mitochondrial DNA is constantly exposed to the mutagenic influence of ROS due to its close proximity to the sites of ROS formation. Therefore, the repair of mitochondrial DNA plays a key role in maintaining mitochondrial function during aging.

## 7. Contribution of Mitochondrially Produced ROS to Age-Related Changes in Signaling Pathways

In addition to the destructive effects, ROS also play a signaling role, launching pathways that activate defense systems and modulate different metabolic pathways [6,11]. A moderate increase of the steady-state ROS levels may increase longevity. For instance, it was observed that naked mole rats have a substantially longer lifespan than other representatives of the same taxonomic rodent family, generating higher steady-state ROS levels and showing higher amounts of oxidatively modified molecules [101,102]. Interestingly, naked mole rats exhibit higher levels of auxiliary antioxidant and xenobiotic detoxification enzymes, such as glutathione-*S*-transferases [103]. Moreover, their mitochondria keep mild depolarization of the inner membrane for a very long period of life [104]. This depolarization was found to be conferred by continuous ATP synthesis due to the immediate expenditure of ATP by hexokinase II and creatine kinase bound to the outer membrane. This expenditure is assumed to be protective because of substantially lowered mitochondrial ROS production [104]. Another study has shown that mitochondria of naked mole rats have the potential to consume more hydrogen peroxide than mitochondria of laboratory mice [102].

Several signaling systems can be regulated by mitochondrially produced ROS. ROS-mediated activation of the transcriptional regulator Nrf2 and the TRPM2 channels were already mentioned above. Protein kinase p38, an activator of transcription factor ATF-2 that is involved in regulation of the cell cycle, is another signaling protein activated by mitochondrial ROS [105]. In the model, proposed by Papaconstantinou and Hsieh, oxidative stress caused by mitochondrially-derived ROS activates the apoptosis signal-regulating kinase 1 (ASK1) via oxidation and dissociation of the bound thioredoxin [106,107]. In turn, ASK1 activates the pro-aging p38 kinase, which was shown to activate senescence-promoting inhibitors of cyclin-dependent kinases, Ink4a and Ink4d (p14/Arf) [106]. Interestingly, the mitochondrial isoform of Ink4d called smArf can induce autophagy [108]. The negative role of ASK1 and p38 signaling in brain aging was confirmed by Hagesawa and colleagues, who found that old mice deficient in ASK1 generated less soluble amyloid β [109].

MitoNEET, the iron-sulfur-cluster-containing redox sensor of the mitochondrial outer membrane discovered in the early 2000s, was found to be important for the maintenance of mitochondrial integrity [110] as it regulates free iron levels, thus preventing the accumulation of iron inside the mitochondrial matrix [111]. In addition, mitoNEET was shown to regulate the gating of the voltage-dependent anion channel (VDAC, also known as porin) [112]. It also helps to restore impaired iron-sulfur clusters of cytosolic aconitase [113]. Interestingly, mice lacking mitoNEET exhibited symptoms of Parkinson’s disease [114]. Besides mitoNEET, cyclophilin D (CypD), the peptidyl prolyl isomerase interacting with several proteins located in the mitochondrial inner membrane (e.g., adenine nucleotide translocator (ANT), ATP synthase and phosphate carrier) is worth mentioning [115]. For a long time, CypD was considered a component of the mitochondrial permeability transition pore (mPTP). Now, there are two putative models for the structure of the mPTP. One model postulates that mPTP consists of ANT bound to CypD in the mitochondrial inner membrane, along with VDAC in the mitochondrial outer membrane. A newer model suggests that CypD is a regulatory but not a mandatory component of the mPTP. According to this model, ATP synthase dimer but not ANT is the inner membrane component of the mPTP, which is regulated by CypD [116]. CypD was shown to contain redox-sensitive cysteine residues that respond to hydrogen peroxide or superoxide anion exposure [115,117]. Interestingly, the expression of CypD was recently found to increase in the mouse brain with age, starting from middle age. Moreover, CypD seems to promote brain aging, causing ROS-mediated opening of mPTP [83]. Therefore, CypD is considered a putative drug target, whose inhibition could extend lifespan and delay neurodegenerative diseases [83]. However, it also seems that the dosage matters, as only partial but not a complete deletion of cypD extended the life- and healthspan in mice [118]. For further detail, we refer the reader to comprehensive reviews included into the recent special issue on mitochondrial oxidative and nitrosative stress as a therapeutic target in diseases [119].

Cumulative data suggest that an experimentally induced decrease in the steady-state ROS level in brains of middle-aged animals delays the age-dependent dysfunction or loss of neurons. Thus, the application of high doses of tocopherol extended the healthspan of rodents by decreasing brain oxidative damage and preventing mitochondrial dysfunction [120]. Similarly, an increase in mitochondrial activity and decrease in the levels of lipid peroxidation products was observed in brains of female mice fed with whey protein [121]. A protective effect of vitamin C in mouse models of Alzheimer’s disease (reviewed by [85]) was also reported. Mitochondrially-targeted antioxidants such as MitoQ and SkQ, ubiquinone and plastoquinone derivatives conjugated with penetrating cation triphenylphosphonium, seem to be even more promising [85,122]. Because of the role of ROS in cell signaling their overall decrease may be ineffective or even detrimental. Antioxidants that specifically target mitochondria may prevent age-dependent loss of the aforementioned mitochondrial proteins without disturbing cell signaling.

## 8. Midlife Activation of the Brain’s Immune System and Its Possible Consequences

Besides intensification of oxidative stress, an organism’s aging is inevitably accompanied by the accumulation of damaged, metabolically modified or displaced self-molecules (e.g., (per)oxidized or nitrated proteins, amyloids, AGEs, damaged nuclear or mitochondrial DNA, small interfering RNAs, cell debris, etc.), which are released into the extracellular space or circulate in bodily fluids. These molecules are perceived by the immune system as danger- or damage-associated molecular patterns (DAMPs). Therefore, aging is accompanied by a chronic low-grade inflammation, summarized by C. Franceschi et al. in the comprehensive term “inflammaging” [123,124]. Inflammaging is a multifactorial systemic process, characterized by increased plasma levels of main proinflammatory cytokines (i.e., interleukin (IL)-1β, IL-6, IL-18, tumor necrosis factor alpha (TNF-α)) and activation of the NF-κB-mediated pathway (Figure 1), and is classically assumed to increase with advancing age [123,124].

In microglia, activation of the NF-κB-mediated pathway by proinflammatory cytokines and DAMPs upregulates the expression of main ROS-producing enzymes (COX2, NOX2 and iNOS), thus stimulating the aberrant production of ROS (Figure 1). Stimulation of microglia by TNF-α, IL-1β, LPS, arachidonic acid or ATP (Figure 1) was shown to generate extracellular ^•^NO and the subsequent stimulation of NOX, resulting in the peroxynitrite formation [14]. Such interplay between iNOS and NOX likely contributes to microglia-mediated neurotoxicity. According to recent data, stimulation of the Toll-Like Receptor (TLR) 4 also supports the activation of NOX in microglia [14], and H_2_O_2_ was shown to activate NF-κB-mediated signaling in peripheral macrophages [12]. While proinflammatory microglia upregulate iNOS, the anti-inflammatory microglia express arginase-1. Since arginase-1 and iNOS compete for the same substrate (L-arginine), their interplay determines the level of ^•^NO production and the resulting intensity of oxidative and/or nitrosative stress. Furthermore, recent transcriptomic studies of microglia identified PTGS2, coding for COX2 enzyme, as one of the most upregulated genes in proinflammatory conditions [14].

Besides stimulating ROS production, the activation of the NF-κB pathway together with an increase in [Ca^2+^]_i_ and mitochondrial ROS upregulates the production and assembly of the NLRP3 inflammasome (Figure 1). The latter, in turn, fuels the microglial IL-1β production. Together with the enhanced microglial production of TNF-α [125], this closes the loop and further exacerbates the aging-related neuroinflammation. The situation is further worsened by aging-induced neuronal hyperactivity [126] and the subsequent release of the excitatory neurotransmitter glutamate [127]. Glutamate activates N-methyl-D-aspartate (NMDA) receptors and increases [Ca^2+^]_i_ in neurons, thus promoting the Ca^2+^/calmodulin-dependent activation of neuronal NOS and the redox imbalance within the brain [14,62]. Moreover, both neuro- and systemic inflammation were recently shown to cause hyperactivity and increased Ca^2+^ signaling in neurons and microglia [64,128,129,130,131].

While previously, the above scenario was believed to describe the last trimester of life, our recent data suggest that many of the described changes begin already at midlife. Indeed, microglia are known to react to an increase in the systemic level of proinflammatory cytokines with an increase in [Ca^2+^]_i_ and under experimental conditions, an increase in microglial Ca^2+^ signaling occurs hours after the induction of inflammation [16,131,132]. At the same time, microglia in middle-aged wild-type mice show a pronounced increase in the intracellular Ca^2+^ signaling, with upregulated frequency, duration and the area under the curve of ongoing Ca^2+^ transients [21,23]. The exacerbated Ca^2+^ signaling is a hallmark of middle-aged microglia and diminishes with advancing age. It promotes the generation of mitochondrial ROS, assembly of the NLRP3 inflammasome and microglial IL-1β production (Figure 1), thus promoting the build-up of the brain’s proinflammatory state. This process is further aggravated by the described increase in mitochondrial activity during midlife, which increases cellular ROS levels and potentiates Ca^2+^ influx through TRPM2 channels (Figure 1), thus further promoting oxidative stress.

Interestingly, the midlife proinflammatory state impacts male and female microglia differently, with male microglia showing a pronounced increase in frequency and female microglia in duration of ongoing Ca^2+^ transients [23]. Furthermore, both the frequency and duration of Ca^2+^ transients did not increase in mice, subjected to calorie restriction (i.e., fed daily with 70% of their respective initial *ad libitum* food intake) between 3 (young adult) and 9–11 (middle-aged) months of age [23]. Besides, calorie restriction was effective in preventing and even reverting other aging-related microglial dysfunctions (e.g., dyscoordination of the microglial process movement towards the source of DAMPs).

Thus, the midlife changes in the inflammatory state of the brain, as well as energy and redox metabolism, are sensed by the brain’s immune cells microglia, in part through intracellular Ca^2+^ signaling. The Ca^2+^ signals govern the executive functions of microglia, including the production of ROS, RNS and proinflammatory cytokines. This likely promotes brain damage but can be decelerated by calorie restriction.

## 9. Conclusions

Reactive oxygen and nitrogen species are crucial contributors to the age-dependent decline in all tissues. Neural tissue, one of the main oxygen consumers in the mammalian body, is especially important in this context. Brain cells, including neurons, astrocytes and microglia, produce ROS through specific enzymatic systems, including complexes of the mitochondrial respiratory chain, multienzyme flavin-containing complexes, monoamine and xanthine oxidases, microglial and endothelial NADPH oxidases and cyclooxygenases in addition to non-enzymatic and potentially uncontrolled mechanisms of ROS production, such as autooxidation of quinones or other aromatic compounds. Nitric oxide produced by nitric oxide synthases powers the conversion of ROS into reactive nitrogen species. Both ROS and RNS play important signaling roles but are also capable of modifying other molecules such as proteins, nucleic and fatty acids, lipids and carbohydrates. The antioxidant system, comprising low molecular mass antioxidants (e.g., tocopherol, ascorbic acid and glutathione) and high molecular mass antioxidants such as enzymes (e.g., catalases, peroxidases, superoxide dismutases) and others, protects cells from potential damage caused by ROS or RNS. Midlife is crucial for the “struggle” between ROS/RNS production and their elimination. Counterintuitively, the age dependence of integrity and operation of many metabolic processes in the neural tissue shows a bell shape rather than a linear decline. This bell-shaped age-dependency is likely caused by metabolic rearrangements, helping cells to cope with increasing levels of ROS/RNS. For instance, at midlife glucose catabolism is rerouted from glycolysis to the PPP, allowing increased production of NADPH, a co-substrate for glutathione and thioredoxin reductases (Figure 3). The powering antioxidant systems by NADPH provides neural tissue with a better defense against ROS but may also intensify ROS production via the action of NADPH oxidases and cyclooxygenases. In turn, mitochondria start using ketone bodies as an energy source. At the same time, midlife seems to be a turning point, after which prooxidative processes start to intensify, despite the metabolic rerouting. In particular, this is connected with a loss in effectiveness of mitochondria, especially improper work of the respiratory chain, which is a ROS generator itself.

Moreover, the increased steady-state levels of ROS, AGEs and RNS, along with the aforementioned ROS-modified self-molecules, activate the organisms’ immune system including brain’s microglia (Figure 2). At midlife, the sensitivity of microglia to these proinflammatory molecules is high, resulting in heightened microglial Ca^2+^ signaling and accelerated production of intra- and extracellular ROS. With advancing age, however, the microglial responsiveness is downregulated, resulting in senescent microglia. Calorie restriction can prevent the deterioration of intracellular Ca^2+^ signaling as well as other functional properties of microglia [23], thus providing a readily available and cost-efficient antiaging lifestyle intervention.

## Data Availability

The data presented in this study are available in the article.

## References

[1] Adam-Vizi V. (2005). Production of Reactive Oxygen Species in Brain Mitochondria: Contribution by Electron Transport Chain and Non-Electron Transport Chain Sources. Antioxid. Redox Signal..

[2] Lushchak V.I. (2015). Free Radicals, Reactive Oxygen Species, Oxidative Stresses and their Classifications. UKR. Biochem. J..

[3] Garaschuk O., Semchyshyn H.M., Lushchak V.I. (2018). Healthy Brain Aging: Interplay between Reactive Species, Inflammation and Energy Supply. Ageing Res. Rev..

[4] Lushchak V.I. (2021). Interplay between Bioenergetics and Oxidative Stress at Normal Brain Aging. Aging as a Result of Increasing Disbalance in the System Oxidative Stress-Energy Provision. Pflugers Arch..

[5] Semchyshyn H. (2021). Is Carbonyl/AGE/RAGE Stress a Hallmark of the Brain Aging?. Pflugers Arch..

[6] Zorov D.B., Juhaszova M., Sollott S.J. (2014). Mitochondrial Reactive Oxygen Species (ROS) and ROS-Induced ROS Release. Physiol. Rev..

[7] Harman D. (1956). Aging: A Theory Based on Free Radical and Radiation Chemistry. J. Gerontol..

[8] McCord J.M., Fridovich I. (1969). Superoxide Dismutase. An Enzymic Function for Erythrocuprein (Hemocuprein). J. Biol. Chem..

[9] Reczek C.R., Chandel N.S. (2015). ROS-Dependent Signal Transduction. Curr. Opin. Cell Biol..

[10] Zandalinas S.I., Mittler R. (2018). ROS-Induced ROS Release in Plant and Animal Cells. Free Radic. Biol. Med..

[11] Sies H., Jones D.P. (2020). Reactive Oxygen Species (ROS) as Pleiotropic Physiological Signalling Agents. Nat. Rev. Mol. Cell Biol..

[12] Simpson D.S.A., Oliver P.L. (2020). ROS Generation in Microglia: Understanding Oxidative Stress and Inflammation in Neurodegenerative Disease. Antioxidants.

[13] Longo V.D., Di Tano M., Mattson M.P., Guidi N. (2021). Intermittent and Periodic Fasting, Longevity and Disease. Nat. Aging.

[14] García-Revilla J., Alonso-Bellido I.M., Burguillos M.A., Herrera A.J., Espinosa-Oliva A.M., Ruiz R., Cruz-Hernández L., García-Domínguez I., Roca-Ceballos M.A., Santiago M. (2019). Reformulating Pro-Oxidant Microglia in Neurodegeneration. J. Clin. Med..

[15] Cepas V., Collino M., Mayo J.C., Sainz R.M. (2020). Redox Signaling and Advanced Glycation Endproducts (AGEs) in Diet-Related Diseases. Antioxidants.

[16] Garaschuk O. (2021). The Role of NLRP3 Inflammasome for Microglial Response to Peripheral Inflammation. Neural Regen. Res..

[17] Tschopp J., Schroder K. (2010). NLRP3 Inflammasome Activation: The Convergence of Multiple Signalling Pathways on ROS Production?. Nat. Rev. Immunol..

[18] Yang S., Qin C., Hu Z.-W., Zhou L.-Q., Yu H.-H., Chen M., Bosco D.B., Wang W., Wu L.-J., Tian D.-S. (2021). Microglia Reprogram Metabolic Profiles for Phenotype and Function Changes in Central Nervous System. Neurobiol. Dis..

[19] Metcalfe N.B., Alonso-Alvarez C. (2010). Oxidative Stress as a Life-History Constraint: The Role of Reactive Oxygen Species in Shaping Phenotypes from Conception to Death: Oxidative Stress as a Life-History Constraint. Funct. Ecol..

[20] Sikora E., Bielak-Zmijewska A., Dudkowska M., Krzystyniak A., Mosieniak G., Wesierska M., Wlodarczyk J. (2021). Cellular Senescence in Brain Aging. Front. Aging Neurosci..

[21] Olmedillas Del Moral M., Asavapanumas N., Uzcátegui N.L., Garaschuk O. (2019). Healthy Brain Aging Modifies Microglial Calcium Signaling In Vivo. Int. J. Mol. Sci..

[22] Yanar K., Simsek B., Çaylı N., Övül Bozkır H., Mengi M., Belce A., Aydin S., Çakatay U. (2019). Caloric Restriction and Redox Homeostasis in Various Regions of Aging Male Rat Brain: Is Caloric Restriction Still Worth Trying Even after Early-Adulthood? Redox Homeostasis and Caloric Restriction in Brain. J. Food Biochem..

[23] Olmedillas Del Moral M., Fröhlich N., Figarella K., Mojtahedi N., Garaschuk O. (2020). Effect of Caloric Restriction on the In Vivo Functional Properties of Aging Microglia. Front. Immunol..

[24] Bayliak M.M., Sorochynska O.M., Kuzniak O.V., Gospodaryov D.V., Demianchuk O.I., Vasylyk Y.V., Mosiichuk N.M., Storey K.B., Garaschuk O., Lushchak V.I. (2021). Middle Age as a Turning Point in Mouse Cerebral Cortex Energy and Redox Metabolism: Modulation by Every-Other-Day Fasting. Exp. Gerontol..

[25] Bayliak M.M., Mosiichuk N.M., Sorochynska O.M., Kuzniak O.V., Sishchuk L.O., Hrushchenko A.O., Semchuk A.O., Pryimak T.V., Vasylyk Y.V., Gospodaryov D.V. (2021). Middle Aged Turn Point in Parameters of Oxidative Stress and Glucose Catabolism in Mouse Cerebellum during Lifespan: Minor Effects of Every-Other-Day Fasting. Biogerontology.

[26] Clarke D.D., Sokoloff L., Siegel G., Agrano B.V., Albers R.W., Molino P.V. (1999). Circulation and Energy Metabolism of the Brain. Basic Neurochemistry.

[27] Hyder F., Rothman D.L., Bennett M.R. (2013). Cortical Energy Demands of Signaling and Nonsignaling Components in Brain Are Conserved across Mammalian Species and Activity Levels. Proc. Natl. Acad. Sci. USA.

[28] Roy C.S., Sherrington C.S. (1890). On the Regulation of the Blood-Supply of the Brain. J. Physiol..

[29] Cipolla M.J. (2009). The Cerebral Circulation. Colloq. Ser. Integr. Syst. Physiol. Mol. Funct..

[30] Moore S.A. (2001). Polyunsaturated Fatty Acid Synthesis and Release by Brain-Derived Cells In Vitro. J. Mol. Neurosci. MN.

[31] Halim N.D., Mcfate T., Mohyeldin A., Okagaki P., Korotchkina L.G., Patel M.S., Jeoung N.H., Harris R.A., Schell M.J., Verma A. (2010). Phosphorylation Status of Pyruvate Dehydrogenase Distinguishes Metabolic Phenotypes of Cultured Rat Brain Astrocytes and Neurons. Glia.

[32] Di Loreto S., Zimmitti V., Sebastiani P., Cervelli C., Falone S., Amicarelli F. (2008). Methylglyoxal Causes Strong Weakening of Detoxifying Capacity and Apoptotic Cell Death in Rat Hippocampal Neurons. Int. J. Biochem. Cell Biol..

[33] Rose J., Brian C., Pappa A., Panayiotidis M.I., Franco R. (2020). Mitochondrial Metabolism in Astrocytes Regulates Brain Bioenergetics, Neurotransmission and Redox Balance. Front. Neurosci..

[34] Debernardi R., Pierre K., Lengacher S., Magistretti P.J., Pellerin L. (2003). Cell-Specific Expression Pattern of Monocarboxylate Transporters in Astrocytes and Neurons Observed in Different Mouse Brain Cortical Cell Cultures. J. Neurosci. Res..

[35] Rafiki A., Boulland J.L., Halestrap A.P., Ottersen O.P., Bergersen L. (2003). Highly Differential Expression of the Monocarboxylate Transporters MCT2 and MCT4 in the Developing Rat Brain. Neuroscience.

[36] Bröer S., Rahman B., Pellegri G., Pellerin L., Martin J.L., Verleysdonk S., Hamprecht B., Magistretti P.J. (1997). Comparison of Lactate Transport in Astroglial Cells and Monocarboxylate Transporter 1 (MCT 1) Expressing *Xenopus laevis* Oocytes. Expression of Two Different Monocarboxylate Transporters in Astroglial Cells and Neurons. J. Biol. Chem..

[37] van Hall G., Strømstad M., Rasmussen P., Jans O., Zaar M., Gam C., Quistorff B., Secher N.H., Nielsen H.B. (2009). Blood Lactate Is an Important Energy Source for the Human Brain. J. Cereb. Blood Flow Metab..

[38] Mattson M.P., Gleichmann M., Cheng A. (2008). Mitochondria in Neuroplasticity and Neurological Disorders. Neuron.

[39] Grimm A., Eckert A. (2017). Brain Aging and Neurodegeneration: From a Mitochondrial Point of View. J. Neurochem..

[40] De Miranda B.R., Rocha E.M., Castro S.L., Greenamyre J.T. (2020). Protection from α-Synuclein Induced Dopaminergic Neurodegeneration by Overexpression of the Mitochondrial Import Receptor TOM20. NPJ Park. Dis..

[41] Herrero-Mendez A., Almeida A., Fernández E., Maestre C., Moncada S., Bolaños J.P. (2009). The Bioenergetic and Antioxidant Status of Neurons Is Controlled by Continuous Degradation of a Key Glycolytic Enzyme by APC/C-Cdh1. Nat. Cell Biol..

[42] Longden T.A., Hill-Eubanks D.C., Nelson M.T. (2016). Ion Channel Networks in the Control of Cerebral Blood Flow. J. Cereb. Blood Flow Metab..

[43] Garthwaite J., Charles S.L., Chess-Williams R. (1988). Endothelium-Derived Relaxing Factor Release on Activation of NMDA Receptors Suggests Role as Intercellular Messenger in the Brain. Nature.

[44] Roman R.J. (2002). P-450 Metabolites of Arachidonic Acid in the Control of Cardiovascular Function. Physiol. Rev..

[45] Hall C.N., Reynell C., Gesslein B., Hamilton N.B., Mishra A., Sutherland B.A., O’Farrell F.M., Buchan A.M., Lauritzen M., Attwell D. (2014). Capillary Pericytes Regulate Cerebral Blood Flow in Health and Disease. Nature.

[46] Santos R.M., Lourenço C.F., Pomerleau F., Huettl P., Gerhardt G.A., Laranjinha J., Barbosa R.M. (2011). Brain Nitric Oxide Inactivation Is Governed by the Vasculature. Antioxid. Redox Signal..

[47] Rolett E.L., Azzawi A., Liu K.J., Yongbi M.N., Swartz H.M., Dunn J.F. (2000). Critical Oxygen Tension in Rat Brain: A Combined (31)P-NMR and EPR Oximetry Study. Am. J. Physiol. Regul. Integr. Comp. Physiol..

[48] Wagenführ L., Meyer A.K., Marrone L., Storch A. (2016). Oxygen Tension Within the Neurogenic Niche Regulates Dopaminergic Neurogenesis in the Developing Midbrain. Stem Cells Dev..

[49] Martí-Fàbregas J., Romaguera-Ros M., Gómez-Pinedo U., Martínez-Ramírez S., Jiménez-Xarrié E., Marín R., Martí-Vilalta J.-L., García-Verdugo J.-M. (2010). Proliferation in the Human Ipsilateral Subventricular Zone after Ischemic Stroke. Neurology.

[50] Ashok B.S., Ajith T.A., Sivanesan S. (2017). Hypoxia-Inducible Factors as Neuroprotective Agent in Alzheimer’s Disease. Clin. Exp. Pharmacol. Physiol..

[51] Watts M.E., Pocock R., Claudianos C. (2018). Brain Energy and Oxygen Metabolism: Emerging Role in Normal Function and Disease. Front. Mol. Neurosci..

[52] Papa S., Skulachev V.P. (1997). Reactive Oxygen Species, Mitochondria, Apoptosis and Aging. Mol. Cell. Biochem..

[53] Büeler H. (2021). Mitochondrial and Autophagic Regulation of Adult Neurogenesis in the Healthy and Diseased Brain. Int. J. Mol. Sci..

[54] Fan L.M., Geng L., Cahill-Smith S., Liu F., Douglas G., Mckenzie C.-A., Smith C., Brooks G., Channon K.M., Li J.-M. (2019). Nox2 Contributes to Age-Related Oxidative Damage to Neurons and the Cerebral Vasculature. J. Clin. Investig..

[55] Vignais P.V. (2002). The Superoxide-Generating NADPH Oxidase: Structural Aspects and Activation Mechanism. Cell. Mol. Life Sci. CMLS.

[56] Kontos H.A., George E. (1985). Brown Memorial Lecture. Oxygen Radicals in Cerebral Vascular Injury. Circ. Res..

[57] Kukreja R.C., Kontos H.A., Hess M.L., Ellis E.F. (1986). PGH Synthase and Lipoxygenase Generate Superoxide in the Presence of NADH or NADPH. Circ. Res..

[58] Terada L.S., Willingham I.R., Rosandich M.E., Leff J.A., Kindt G.W., Repine J.E. (1991). Generation of Superoxide Anion by Brain Endothelial Cell Xanthine Oxidase. J. Cell. Physiol..

[59] Minaev B.F. (2019). How Cofactor-Free Oxygenases Can Overcome Spin Prohibition in Substrates Oxygenation by Dioxygen. Chem. Phys..

[60] Hanschmann E.-M., Godoy J.R., Berndt C., Hudemann C., Lillig C.H. (2013). Thioredoxins, Glutaredoxins, and Peroxiredoxins—Molecular Mechanisms and Health Significance: From Cofactors to Antioxidants to Redox Signaling. Antioxid. Redox Signal..

[61] Jones D.P. (2015). Redox Theory of Aging. Redox Biol..

[62] Asiimwe N., Yeo S.G., Kim M.-S., Jung J., Jeong N.Y. (2016). Nitric Oxide: Exploring the Contextual Link with Alzheimer’s Disease. Oxid. Med. Cell. Longev..

[63] Brawek B., Garaschuk O. (2013). Microglial Calcium Signaling in the Adult, Aged and Diseased Brain. Cell Calcium.

[64] Brawek B., Garaschuk O. (2017). Monitoring In Vivo Function of Cortical Microglia. Cell Calcium.

[65] Garaschuk O., Verkhratsky A. (2019). Physiology of Microglia. Methods Mol. Biol..

[66] Brookes P.S., Yoon Y., Robotham J.L., Anders M.W., Sheu S.-S. (2004). Calcium, ATP, and ROS: A Mitochondrial Love-Hate Triangle. Am. J. Physiol. Cell Physiol..

[67] Wang L., Negro R., Wu H. (2020). TRPM2, Linking Oxidative Stress and Ca2+ Permeation to NLRP3 Inflammasome Activation. Curr. Opin. Immunol..

[68] Sorochynska O.M., Bayliak M.M., Gospodaryov D.V., Vasylyk Y.V., Kuzniak O.V., Pankiv T.M., Garaschuk O., Storey K.B., Lushchak V.I. (2019). Every-Other-Day Feeding Decreases Glycolytic and Mitochondrial Energy-Producing Potentials in the Brain and Liver of Young Mice. Front. Physiol..

[69] Calabrese V., Cornelius C., Trovato A., Cavallaro M., Mancuso C., Di Rienzo L., Condorelli D., De Lorenzo A., Calabrese E.J. (2010). The Hormetic Role of Dietary Antioxidants in Free Radical-Related Diseases. Curr. Pharm. Des..

[70] Bayliak M.M., Abrat O.B., Deng H. (2020). Role of Nrf2 in Oxidative and Inflammatory Processes in Obesity and Metabolic Diseases. Nrf2 and Its Modulation in Inflammation.

[71] Chorley B.N., Campbell M.R., Wang X., Karaca M., Sambandan D., Bangura F., Xue P., Pi J., Kleeberger S.R., Bell D.A. (2012). Identification of Novel NRF2-Regulated Genes by ChIP-Seq: Influence on Retinoid X Receptor Alpha. Nucleic Acids Res..

[72] Turell L., Zeida A., Trujillo M. (2020). Mechanisms and Consequences of Protein Cysteine Oxidation: The Role of the Initial Short-Lived Intermediates. Essays Biochem..

[73] Forman H.J., Davies M.J., Krämer A.C., Miotto G., Zaccarin M., Zhang H., Ursini F. (2017). Protein Cysteine Oxidation in Redox Signaling: Caveats on Sulfenic Acid Detection and Quantification. Arch. Biochem. Biophys..

[74] Alcock L.J., Perkins M.V., Chalker J.M. (2018). Chemical Methods for Mapping Cysteine Oxidation. Chem. Soc. Rev..

[75] Pomatto L.C.D., Davies K.J.A. (2017). The Role of Declining Adaptive Homeostasis in Ageing. J. Physiol..

[76] Meng J., Lv Z., Qiao X., Li X., Li Y., Zhang Y., Chen C. (2017). The Decay of Redox-Stress Response Capacity Is a Substantive Characteristic of Aging: Revising the Redox Theory of Aging. Redox Biol..

[77] Baker D.J., Peleg S. (2017). Biphasic Modeling of Mitochondrial Metabolism Dysregulation during Aging. Trends Biochem. Sci..

[78] Navarro A., Boveris A. (2004). Rat Brain and Liver Mitochondria Develop Oxidative Stress and Lose Enzymatic Activities on Aging. Am. J. Physiol. Regul. Integr. Comp. Physiol..

[79] Navarro A., López-Cepero J.M., Bández M.J., Sánchez-Pino M.-J., Gómez C., Cadenas E., Boveris A. (2008). Hippocampal Mitochondrial Dysfunction in Rat Aging. Am. J. Physiol. Regul. Integr. Comp. Physiol..

[80] Boveris A., Navarro A. (2008). Brain Mitochondrial Dysfunction in Aging. IUBMB Life.

[81] Yao J., Hamilton R.T., Cadenas E., Brinton R.D. (2010). Decline in Mitochondrial Bioenergetics and Shift to Ketogenic Profile in Brain during Reproductive Senescence. Biochim. Biophys. Acta.

[82] Li H., Kumar Sharma L., Li Y., Hu P., Idowu A., Liu D., Lu J., Bai Y. (2013). Comparative Bioenergetic Study of Neuronal and Muscle Mitochondria during Aging. Free Radic. Biol. Med..

[83] Gauba E., Guo L., Du H. (2017). Cyclophilin D Promotes Brain Mitochondrial F1FO ATP Synthase Dysfunction in Aging Mice. J. Alzheimers Dis..

[84] Klosinski L.P., Yao J., Yin F., Fonteh A.N., Harrington M.G., Christensen T.A., Trushina E., Brinton R.D. (2015). White Matter Lipids as a Ketogenic Fuel Supply in Aging Female Brain: Implications for Alzheimer’s Disease. EBioMedicine.

[85] Mi Y., Qi G., Brinton R.D., Yin F. (2021). Mitochondria-Targeted Therapeutics for Alzheimer’s Disease: The Good, the Bad, the Potential. Antioxid. Redox Signal..

[86] Norwitz N.G., Hu M.T., Clarke K. (2019). The Mechanisms by Which the Ketone Body D-β-Hydroxybutyrate May Improve the Multiple Cellular Pathologies of Parkinson’s Disease. Front. Nutr..

[87] Kirkwood T.B.L. (2005). Understanding the Odd Science of Aging. Cell.

[88] Diaz F., Garcia S., Padgett K.R., Moraes C.T. (2012). A Defect in the Mitochondrial Complex III, but Not Complex IV, Triggers Early ROS-Dependent Damage in Defined Brain Regions. Hum. Mol. Genet..

[89] Maranzana E., Barbero G., Falasca A.I., Lenaz G., Genova M.L. (2013). Mitochondrial Respiratory Supercomplex Association Limits Production of Reactive Oxygen Species from Complex I. Antioxid. Redox Signal..

[90] Pinto M., Vempati U.D., Diaz F., Peralta S., Moraes C.T. (2019). Ablation of Cytochrome c in Adult Forebrain Neurons Impairs Oxidative Phosphorylation Without Detectable Apoptosis. Mol. Neurobiol..

[91] Reichart G., Mayer J., Zehm C., Kirschstein T., Tokay T., Lange F., Baltrusch S., Tiedge M., Fuellen G., Ibrahim S. (2019). Mitochondrial Complex IV Mutation Increases Reactive Oxygen Species Production and Reduces Lifespan in Aged Mice. Acta Physiol..

[92] Lopez-Fabuel I., Le Douce J., Logan A., James A.M., Bonvento G., Murphy M.P., Almeida A., Bolaños J.P. (2016). Complex I Assembly into Supercomplexes Determines Differential Mitochondrial ROS Production in Neurons and Astrocytes. Proc. Natl. Acad. Sci. USA.

[93] Lushchak O.V., Piroddi M., Galli F., Lushchak V.I. (2014). Aconitase Post-Translational Modification as a Key in Linkage between Krebs Cycle, Iron Homeostasis, Redox Signaling, and Metabolism of Reactive Oxygen Species. Redox Rep. Commun. Free Radic. Res..

[94] De Pinto V., Reina S., Gupta A., Messina A., Mahalakshmi R. (2016). Role of Cysteines in Mammalian VDAC Isoforms’ Function. Biochim. Biophys. Acta.

[95] Yang X., Wu J., Jing S., Forster M.J., Yan L.-J. (2018). Mitochondrial Protein Sulfenation during Aging in the Rat Brain. Biophys. Rep..

[96] Chung W.-G., Miranda C.L., Maier C.S. (2008). Detection of Carbonyl-Modified Proteins in Interfibrillar Rat Mitochondria Using N’-Aminooxymethylcarbonylhydrazino-D-Biotin as an Aldehyde/Keto-Reactive Probe in Combination with Western Blot Analysis and Tandem Mass Spectrometry. Electrophoresis.

[97] Wu J., Luo X., Yan L.-J. (2015). Two Dimensional Blue Native/SDS-PAGE to Identify Mitochondrial Complex I Subunits Modified by 4-Hydroxynonenal (HNE). Front. Physiol..

[98] Graziewicz M.A., Day B.J., Copeland W.C. (2002). The Mitochondrial DNA Polymerase as a Target of Oxidative Damage. Nucleic Acids Res..

[99] Niedernhofer L.J., Gurkar A.U., Wang Y., Vijg J., Hoeijmakers J.H.J., Robbins P.D. (2018). Nuclear Genomic Instability and Aging. Annu. Rev. Biochem..

[100] Kazak L., Reyes A., Holt I.J. (2012). Minimizing the Damage: Repair Pathways Keep Mitochondrial DNA Intact. Nat. Rev. Mol. Cell Biol..

[101] Andziak B., O’Connor T.P., Qi W., DeWaal E.M., Pierce A., Chaudhuri A.R., Van Remmen H., Buffenstein R. (2006). High Oxidative Damage Levels in the Longest-Living Rodent, the Naked Mole-Rat. Aging Cell.

[102] Munro D., Baldy C., Pamenter M.E., Treberg J.R. (2019). The Exceptional Longevity of the Naked Mole-Rat May Be Explained by Mitochondrial Antioxidant Defenses. Aging Cell.

[103] Lewis K.N., Wason E., Edrey Y.H., Kristan D.M., Nevo E., Buffenstein R. (2015). Regulation of Nrf2 Signaling and Longevity in Naturally Long-Lived Rodents. Proc. Natl. Acad. Sci. USA.

[104] Vyssokikh M.Y., Holtze S., Averina O.A., Lyamzaev K.G., Panteleeva A.A., Marey M.V., Zinovkin R.A., Severin F.F., Skulachev M.V., Fasel N. (2020). Mild Depolarization of the Inner Mitochondrial Membrane Is a Crucial Component of an Anti-Aging Program. Proc. Natl. Acad. Sci. USA.

[105] Hsieh C.-C., Kuro-o M., Rosenblatt K.P., Brobey R., Papaconstantinou J. (2010). The ASK1-Signalosome Regulates P38 MAPK Activity in Response to Levels of Endogenous Oxidative Stress in the Klotho Mouse Models of Aging. Aging.

[106] Papaconstantinou J., Hsieh C.-C. (2010). Activation of Senescence and Aging Characteristics by Mitochondrially Generated ROS: How Are They Linked?. Cell Cycle.

[107] Saitoh M., Nishitoh H., Fujii M., Takeda K., Tobiume K., Sawada Y., Kawabata M., Miyazono K., Ichijo H. (1998). Mammalian Thioredoxin Is a Direct Inhibitor of Apoptosis Signal-Regulating Kinase (ASK) 1. EMBO J..

[108] Mammucari C., Rizzuto R. (2010). Signaling Pathways in Mitochondrial Dysfunction and Aging. Mech. Ageing Dev..

[109] Hasegawa Y., Toyama K., Uekawa K., Ichijo H., Kim-Mitsuyama S. (2018). Role of ASK1/P38 Cascade in a Mouse Model of Alzheimer’s Disease and Brain Aging. J. Alzheimers Dis..

[110] Furihata T., Takada S., Kakutani N., Maekawa S., Tsuda M., Matsumoto J., Mizushima W., Fukushima A., Yokota T., Enzan N. (2021). Cardiac-Specific Loss of MitoNEET Expression Is Linked with Age-Related Heart Failure. Commun. Biol..

[111] Kusminski C.M., Holland W.L., Sun K., Park J., Spurgin S.B., Lin Y., Askew G.R., Simcox J.A., McClain D.A., Li C. (2012). MitoNEET-Driven Alterations in Adipocyte Mitochondrial Activity Reveal a Crucial Adaptive Process That Preserves Insulin Sensitivity in Obesity. Nat. Med..

[112] Lipper C.H., Stofleth J.T., Bai F., Sohn Y.-S., Roy S., Mittler R., Nechushtai R., Onuchic J.N., Jennings P.A. (2019). Redox-Dependent Gating of VDAC by MitoNEET. Proc. Natl. Acad. Sci. USA.

[113] Ferecatu I., Gonçalves S., Golinelli-Cohen M.-P., Clémancey M., Martelli A., Riquier S., Guittet E., Latour J.-M., Puccio H., Drapier J.-C. (2014). The Diabetes Drug Target MitoNEET Governs a Novel Trafficking Pathway to Rebuild an Fe-S Cluster into Cytosolic Aconitase/Iron Regulatory Protein 1. J. Biol. Chem..

[114] Geldenhuys W.J., Benkovic S.A., Lin L., Yonutas H.M., Crish S.D., Sullivan P.G., Darvesh A.S., Brown C.M., Richardson J.R. (2017). MitoNEET (CISD1) Knockout Mice Show Signs of Striatal Mitochondrial Dysfunction and a Parkinson’s Disease Phenotype. ACS Chem. Neurosci..

[115] Amanakis G., Murphy E. (2020). Cyclophilin D: An Integrator of Mitochondrial Function. Front. Physiol..

[116] Carraro M., Checchetto V., Szabó I., Bernardi P. (2019). F-ATP Synthase and the Permeability Transition Pore: Fewer Doubts, More Certainties. FEBS Lett..

[117] Linard D., Kandlbinder A., Degand H., Morsomme P., Dietz K.-J., Knoops B. (2009). Redox Characterization of Human Cyclophilin D: Identification of a New Mammalian Mitochondrial Redox Sensor?. Arch. Biochem. Biophys..

[118] Vereczki V., Mansour J., Pour-Ghaz I., Bodnar I., Pinter O., Zelena D., Oszwald E., Adam-Vizi V., Chinopoulos C. (2017). Cyclophilin D Regulates Lifespan and Protein Expression of Aging Markers in the Brain of Mice. Mitochondrion.

[119] Marí M., Colell A. (2021). Mitochondrial Oxidative and Nitrosative Stress as a Therapeutic Target in Diseases. Antioxidants.

[120] Navarro A., Bandez M.J., Lopez-Cepero J.M., Gómez C., Boveris A. (2011). High Doses of Vitamin E Improve Mitochondrial Dysfunction in Rat Hippocampus and Frontal Cortex upon Aging. Am. J. Physiol. Regul. Integr. Comp. Physiol..

[121] Shertzer H.G., Krishan M., Genter M.B. (2013). Dietary Whey Protein Stimulates Mitochondrial Activity and Decreases Oxidative Stress in Mouse Female Brain. Neurosci. Lett..

[122] Apostolova N., Victor V.M. (2015). Molecular Strategies for Targeting Antioxidants to Mitochondria: Therapeutic Implications. Antioxid. Redox Signal..

[123] Franceschi C., Bonafè M., Valensin S., Olivieri F., De Luca M., Ottaviani E., De Benedictis G. (2000). Inflamm-Aging. An Evolutionary Perspective on Immunosenescence. Ann. N. Y. Acad. Sci..

[124] Franceschi C., Garagnani P., Vitale G., Capri M., Salvioli S. (2017). Inflammaging and “Garb-Aging”. Trends Endocrinol. Metab..

[125] Santello M., Volterra A. (2012). TNFα in Synaptic Function: Switching Gears. Trends Neurosci..

[126] Maier F.C., Wehrl H.F., Schmid A.M., Mannheim J.G., Wiehr S., Lerdkrai C., Calaminus C., Stahlschmidt A., Ye L., Burnet M. (2014). Longitudinal PET-MRI Reveals β-Amyloid Deposition and RCBF Dynamics and Connects Vascular Amyloidosis to Quantitative Loss of Perfusion. Nat. Med..

[127] Lerdkrai C., Asavapanumas N., Brawek B., Kovalchuk Y., Mojtahedi N., Olmedillas Del Moral M., Garaschuk O. (2018). Intracellular Ca2+ Stores Control In Vivo Neuronal Hyperactivity in a Mouse Model of Alzheimer’s Disease. Proc. Natl. Acad. Sci. USA.

[128] Brawek B., Schwendele B., Riester K., Kohsaka S., Lerdkrai C., Liang Y., Garaschuk O. (2014). Impairment of In Vivo Calcium Signaling in Amyloid Plaque-Associated Microglia. Acta Neuropathol..

[129] Busche M.A., Eichhoff G., Adelsberger H., Abramowski D., Wiederhold K.-H., Haass C., Staufenbiel M., Konnerth A., Garaschuk O. (2008). Clusters of Hyperactive Neurons near Amyloid Plaques in a Mouse Model of Alzheimer’s Disease. Science.

[130] Odoj K., Brawek B., Asavapanumas N., Mojtahedi N., Heneka M.T., Garaschuk O. (2021). In Vivo Mechanisms of Cortical Network Dysfunction Induced by Systemic Inflammation. Brain. Behav. Immun..

[131] Riester K., Brawek B., Savitska D., Fröhlich N., Zirdum E., Mojtahedi N., Heneka M.T., Garaschuk O. (2020). In Vivo Characterization of Functional States of Cortical Microglia during Peripheral Inflammation. Brain. Behav. Immun..

[132] Brawek B., Skok M., Garaschuk O. (2021). Changing Functional Signatures of Microglia along the Axis of Brain Aging. Int. J. Mol. Sci..

